# Her Heart's Mistake

**Published:** 1889-06-01

**Authors:** E. D. Cumming

**Affiliations:** Author of "An Ordeal by Silence"


### Her Heart's Mistake.

By E. D. Cumming, Author of "An Ordeal by Silence."

*(Continued from page 124.)*

### Chapter VIII.—Just in Time.

"Oh, for the monsoon," was the cry from white man and black alike that summer in the northern plains. The scorching sun still reigned unchallenged in a brazen sky, taking from all things green the life it had bestowed in kindlier days. The village tanks had fallen lower and lower, until now they held a last half-inch of stagnant water, so green and nauseous that even the gaunt pariahs sniffed, and turned from it with their thirst unquenched. The shallow wells had yielded up thir last drop, and the bullocks lay idle in the shade, their necks freed from the yoke until the rains should send them back to work. The lean ryots looked in dismay upon the parched and sun-cracked fields, and then at the starving plough cattle, whose bones were on the verge of cutting through their hides. The cunning grey-headed crows perched silent in the banyans with beaks agape, and drooping wings, watching for a chance to steal a drink from the earthen ghurras of water the women brought on their heads from the canal; for they, the last of the animal world to suffer, now felt the heat. The mighty Jumna was a narrow thread in the desert of sand which formed its bed when the Himalayan torrents sent down their floods. The canals were running low between their bunds, for the engineer in charge of the irrigation works had been warned to economise the water. District officers were looking grave and anxious, for whispers of famine had been heard from Simla, where Indian legislators were shaking their heads over hundreds of reports, all of which had the same story of drought to tell. They knew what famine meant; they had seen it ravage the land at its pleasure before, secured from human hindrance by the monarchy of the sun; they had seen it leave the dingy mud-built villages tenanted by the unburied skeletons of man and beast, which jackal and vulture picked clean undisturbed. And while natives watched with straining eyes for the clouds to appear on the horizon, the whites turned eagerly to the columns of the *Pioneer* to seek that telegram from Calicut or Mangalore, whose few words "Monsoon broke here to-day," meant so much for all.

It was the middle of July, but not a drop of rain had fallen yet for many hundred miles south of Meerut, and the residents of that station knew that when it did break they must watch the monsoon's progress up the coast for many days ere it touch the boundary of the far north-west. A heavy blow had fallen upon Meerut: a blow that wrought havoc in the hospitals. The factory had broken down, and for five days not a morsel of ice had been procuracle for money. Life at home can furnish no just parallel to this disaster; the ice-chest in Eastern houseeholds is the most important piece of furniture the bungalow contains, and only Anglo-Indians can appreciate the feelings provoked by the water-carrier's message "Can get no ice," when he returns empty-handed from his daily errand to the factory.

George Bairdsley had reached the seventh day of his fever, which until now had shown no signs of abatement. His temperature rose a little one day, and fell again the next, only to rise once more; but while it flickered thus, it never sank below the mark at which the Doctor first found it.

"I feel awfully queer to-night, Kitty," he said to his fiancée that evening. "I don't think I can survive this much longer."

Kate left her chair, and, coming to his bedside, placed her cool hand upon his forehead, while she looked anxiously in his face. A week of fever had worked a change in him. His cheeks were grey and sunken under the thick bristly growth which no razor had touched since his accident, and his eyes were bright and glassy. She went to call her father, who was reading outside the door in the verandah.

"Papa, come here and look at George," she said, in a low voice.

The Doctor threw aside his book and obeyed the summons at once, for he had been growing uneasy about the patient for the last day or two, though he had been careful to conceal it from his daughter. He took Bairdsley's wrist and held it between his fingers for a few moments, then let it go, and called to the servants to bring blankets. The invalid made no protest this time. He was weak and unnerved, and would have tolerated anything his medical adviser saw fit to propose. They rolled him up, and Dr. Harrison sat down on the edge of the bed watching him silently. Suddenly Bairdsley became conscious of a new sensation, and knew that a few beads of perspiration were bursting forth upon his brow and neck; they grew larger and larger and trickled down on to the pillow, and then he felt as though he were literally melting away bodily through every pore. It startled him by its unlooked-for violence, but he rather enjoyed the feeling than otherwise, and when his drenched clothing and sheets had been changed, his spirits rose, a fierce appetite seized him, and he begged with eagerness an immediate meal. The fever had spent itself and had passed away for a time.

The night was oppressive and close, and he kept the punkah coolie at his post until daylight, waking when the motionless air told him that the drowsy servant had gone to sleep, and slumbering again until the fitful jerking stopped once more. He had only a brief respite, however. Twenty-four hours later the fever laid its hand upon him again, and he tossed and moaned as miserably as ever. To make matters worse, the evening brought with it a dust-storm, and every crack of the venetians had to be closed. The wind upon whose wings the dust clouds were borne was hardly perceptible, but the yellowy-grey mist itself was painfully manifest. It filled his eyes and nostrils, gritted between his teeth, and he felt it between his fingers and on his pillow. They might close the doors as tightly as they would, but the fine dust made its way in and settled thickly upon bedclothes, furniture, and floor. And Bairdsley groaned in his wretchedness and cursed his folly in coming to such a country as this, where the breaking of a pinion-wheel could cut off the one luxury which made existence bearable even to those in health.

"Well, Tops, you are looking very glum!" said Captain Phelps to that young officer as he came into the former's bungalow one morning during Bairdsley's illness. "Anything gone wrong with the pony?"

"No, *it's* all right," answered Mr. Wentworth. "Phelps, I've been thinking that I shall have to exchange."

"Exchange!" exclaimed his senior in astonishment. "What has induced you to think of leaving the regiment; you have scarcely put in six months with it."

"I feel that I had better go; I really can't stay on now," said Tops, with a profound sigh.

There was no mistaking the import of that sigh, and Captain Phelps took his cheroot from his lips to blow a succession of blue rings towards the ceiling before he replied.

"I wouldn't talk about exchanging, Tops; take my word you will soon get over it."

Mr. Wentworth shook his head; Captain Phelps's promptness in divining his feelings inclined him to be confidential, and it was a relief to be able to unburden his lovesick soul to a sympathetic auditor.

"You see, Bairdsley's being in the regiment makes it so hard for me. To see her every day, and have to treat her like an ordinary friend. I really couldn't stand it."

Captain Phelps smiled slightly. He was guide, philosopher, and friend by tacit consent to all young officers of the 110th in their difficulties; which, however, were generally of a more prosaic nature: money, for instance. He repressed the smile, and roused himself to soothe the disconsolate Tops.

"Is this the first time you have ever felt anything of—of this kind, Wentworth?"

"Yes, the first; but I know that there never can be another," answered the youth, gravely.

"How old are you, if you don't mind my asking?"

"Not at all; I was twenty last December."

"Ah! Twenty last December. Well, Tops, by the time I was twenty, I had given away my young affections three separate and distinct times to that number of girls. I loved them all with consuming passion by turns, and broke my heart irreparably when each one refused me. I got over the second disappointment with less difficulty than the first, and the third more easily than the second; because I was getting used to the thing. I'm not chaffing you," said the Captain, as Tops grinned a strained perfunctory grin at what he considered ill-time pleasantry. "I am telling you the sober truth. Now, tell me, Wentworth, when did you first feel gone on Miss Harrison?"

"The very moment I saw her; and I have never had a thought for any woman since."

"Quite so; you and I are constituted in much the same way. I used to fall in love at first sight, too. Depend upon it, Wentworth, the fellow who loses his heart most easily gets it back with the least pain, though I grant you it may be sore enough for a while to make him close his eyes and say the world's a howling wilderness."

"Do you think so, really?" asked Tops, by no means certain whether to regard his senior's utterances as those of a philosopher or a flirt.

"I don't imagine I'm very different to other men, and I am quite sure of it. Now, you set yourself to get all the fun you can, and don't waste your time bemoaning the impossible. You could not have married Miss Harrison, Tops, even had the lady been willing."

"We might have waited," sighed Mr. Wentworth; "but, of course, it's useless to discuss it now. I should get over the business sooner if I exchanged and went away from her, though."

"You'd make a big mistake to exchange. You get on very well with us all, and we like you. Your regiment's your home. But perhaps you thought of going to Staff Corps?"

"No; my mother wouldn't like it, and I owe her everything."

Captain Phelps nodded approval. "You're quite right to consider her, but let me advise you strongly to give up this idea of leaving the regiment, where you have plenty of friends. Friends are more easy to make than to keep, Tops, and easier to lose than either. Don't throw away your best ones for the sake of a shadow."

Mr. Wentworth played with the rowel of his spur for a few minutes in silence, and his mentor smoked on placidly.

"Perhaps Bairdsley will exchange to a corps at home, or even chuck the service altogether when he marries," continued Captain Phelps. "He is a rich man, and I know he's disgusted with India already. Better hold on where you are for a bit and see what he intends to do."

The sagacious Phelps knew that a little delay would save his junior from taking the step he proposed, and was also aware that such counsel would be more readily acted upon than his definite recommendation to give up the idea of exchanging altogether.

"I might do that," said Mr. Wentworth, pensively.

"Of course you are down on your luck just now; but wait until the cold weather comes and see if you don't cast away dull care then. You haven't seen the Miss Allertons, have you?"

"The General's daughters? No; but what about them?"

"Why surely some of our fellows must have told you what the Miss Allertons are like. You'll get on with them: the General is very nice to young fellows too, and so is Mrs. Allerton. Their bungalow is the pleasantest in the station during the winter."

Mr. Wentworth flushed with momentary anger. It was revolting to his feelings to be told that there were girls in India, aye, in Meerut, who might supplant Kate Harrison in his bosom. But Captain Phelps spoke so kindly, and was so evidently trying to bring him the consolation his own lights suggested, that his annoyance vanished.

"Mrs. Tenterdale did tell me something about them once," he said, carelessly.

"Well, wait till you see them. Good thing to have a General for a father-in-law, Tops. He would be safe to appoint you his A.D.C., and there's two hundred a month staff pay straight off the reel. You'd always wear spurs in uniform too, Tops. Think of that!"

Mr. Wentworth was not disposed to weigh these infinite possibilities at the present moment; and he thought Captain Phelps was carrying his advice too far when he actually summed up the advantages matrimony with one of the General's daughters would imply. He sighed very heavily and stood up to go.

"Where are you off too?" enquired his friend.

"I'm just going across to ask after Bairdsley," replied Mr. Wentworth.

Captain Phelps bade him good morning and saw him depart, with a shrug and a smile.

"Oh, the pretty candle!—the unlucky moth!" he said to himself. "That candle wouldn't let the silliest moth burn its wings if she could help it, but she can't. She must shine, and the moths will flutter round. I wonder what Tops thought of my confession, that I had loved three girls before I was twenty," soliloquised the Captain; "I might have told him with equal truth that I had had a new one every year or eighteen months ever since—it would have been the bare fact. Very odd; it seems to be a necessity of my nature to be always loving someone; and as soon as I get over the loss of one girl I catch myself looking about for another. I love each one for the time being with all the strength of my being; and I believe that if by any accident I married, I should be as good a husband as man could be. I fancy that I have an eye for beauty—just as some people have an ear for music. A generous capacity for admiration has its advantages. The man who is most easily pleased has the best chance of happiness in life. It's a sad thing for a fellow's whole career to be blighted by a disappointment in love. I can't think that the theoretical lover, who gives his affections once and for all, can be a very broad-minded individual; and the heart must be peculiarly constructed that can find but one resting-place to suit it among the host it comes in contact with. It's an illiberal way of loving, and, to my mind, savours more of obstinacy than the noble attributes it's popularly held to denote. What is the good, where can be the satisfaction of keeping your door everlastingly open for somebody whom you know won't come in? I should like to analyse the process of unconscious reasoning by which a fellow comes to the conclusion that he loves a girl. I can't do it, and heaven knows I've had lots of practice. No novelist has ever done it, but perhaps the language of the future will consist of words which express feeling as well as thought. I hope it won't be a literary development of my day; novels are stupid enough already."

Captain Phelps's thoughts at this point were diverted by the reappearance of Mr. Wentworth coming along the compound path, and he ceased his reflections upon the tender passion to ask what brought him back.

"Bairdsley has had another attack of fever, and his temperature is up to a hundred and five. They are getting frightened about him at the corner bungalow; they've put his bed right up against the kuskus tattie, and have got two coolies working the thermantidote like mad behind it."

"By Jove, that's bad, Wentworth. Whom did you see?"

"Miss Harrison. She looked awfully white, but was as cool and collected as possible, directing the servants about. I've just sent away a telegram for her to Delhi, ordering two hundredweight of ice."

"You didn't see Bairdsley, I suppose?"

"No; I only went into the verandah, and saw Miss Harrison there."

"I wonder what brought on this second attack so soon," said Captain Phelps.

"I believe he hurt his wrist somehow; Miss Harrison didn't say distinctly."

"Tried to throw something at his punkah-wallah, probably," said Phelps to himself; "but he's paying a high price for the pleasure if that was it."

It was not a kindly surmise, but, unfortunately, it happened to be quite correct. Captain Phelps's knowledge of his late chum's practices had enabled him to hit upon the solution, which only Dr. Harrison and the recalcitrant punkah-wallah besides could have given.

Captain Bairdsley's popularity among his friends brought many callers to the corner bungalow that evening to make enquiries about him when it became known that he had succumbed to another attack of fever. No one saw the invalid or Kate Harrison, who, with her aunt, was in close attendance upon him. Anxiety for him, and pity for his sufferings, added to the recollection of those cruel doubts which she had allowed herself to entertain the other day, had caused Kate to devote herself more exclusively than ever to nursing him; and, had her father permitted it, she would have remained up all night, dressed, in readiness to answer his faintest call.

He was delirious during the ensuing night, and raved wildly about Kate, whose name he repeated over and over again, as if endeavouring to impress it upon some imaginary listener. Mrs. Brent was sitting with him at the time, and listened tearfully to the intense earnestness with which he presently declared that he must marry her niece, that nothing under heaven should stop him. She strove to calm him by assurances that no one would ever attempt to come between them, but her words fell on deaf ears. He paused in his wanderings while she spoke, but as soon as the sound of her voice ceased he recommenced his declarations of freedom to do what he chose in regard to Kate Harrison. He must marry her, and he would.

Kate was sitting in the verandah, her mind full of these thoughts, on the evening of the day upon which it had been found necessary to procure ice from Delhi. She had not been allowed to enter her lover's apartment since the morning, and was growing nervous and unstrung.

"How is he, papa?" she asked again, as the Doctor came out of the room.

"Much the same. What time did you send off the message for ice?"

"About half-past eight this morning. I got the railway guide, and made out that it would be here by about five at the latest."

"It's near six now, so you may begin to think about sending someone to the station," said the Doctor. The mahli can go; he's been fooling about with a watering-pot for the last three months, and not doing a stroke of work beyond that."

So the gardener was called from his own sphere of uselessness, and sent up to the railway station with injunctions to hire coolies and bring down the box, which the station-master would give him, the instant the Delhi train arrived.

Either the train was late or the coolies unconscionably slow, for three times the Doctor pushed aside the purdah and asked if the ice had not come. His anxiety infected Kate, and she gave way to her fears, walking up and down the verandah praying for the speedy arrival of the ice, upon which that precious life might depend.

At last the monotonous chant of coolies carrying a load became audible, and four half-naked men trotted into the compound bearing the longed-for box slung upon a pole. Half the servants in the house were immediately set to work to open it, and break the transparent slabs to pieces; and so busy was Kate in seeing to this that she did not hear the remark with which her father received the news that the ice had come, "Just in time; all the ice in the Arctic Circle would not have been worth a pice in another half hour. His temperature is 106 now."

However, there it was: in time to save George Bairdsley's life.

*(To be continued.)*

